# Editorial: Dust mite allergy: immunological mechanisms, environmental impact, genetic susceptibility, diagnosis, therapeutic strategies & preventive measures

**DOI:** 10.3389/falgy.2026.1909730

**Published:** 2026-07-03

**Authors:** Arghya Laha, Leticia Martín-Cruz, Pongsakorn Tantilipikorn

**Affiliations:** 1 Hapten Biosystems Private Limited, Bhiwandi, Maharashtra, India; 2 Department of Biochemistry and Molecular Biology, School of Chemistry, Complutense University, Madrid, Spain; 3 Department of Biochemistry and Molecular Biology, School of Pharmacy, Complutense University, Madrid, Spain; 4 Center of Research Excellence in Allergy & Immunology, Faculty of Medicine Siriraj Hospital, Mahidol University, Bangkok, Thailand

**Keywords:** Der pII, *Dermatophagoides farinae*, *Dermatophagoides pteronyssinus*, dust mite hypersensitivity, IgE, molecular diagnostics, skin prick test

House dust mite (HDM) allergy remains one of the most prevalent allergic disorders worldwide and is a major contributor to allergic rhinitis (AR), asthma, and atopic dermatitis (AD) (1, 2). Sensitization is primarily driven by exposure to allergenic proteins derived from the feces, secretions, and shed body parts of *Dermatophagoides pteronyssinus* and *Dermatophagoides farina*e, which thrive in warm and humid indoor environments (3). The resulting IgE-mediated immune response imposes a substantial global health burden and continues to rise in prevalence, particularly in rapidly urbanizing regions (4). HDM sensitization occurs in more than 50% of allergic individuals living in HDM-rich environments, with an estimated 65–130 million people worldwide experiencing HDM allergy (5). Recent advances in allergy research have expanded our understanding of HDM sensitization, disease mechanisms, and clinical phenotypes. While conventional diagnostic approaches rely on skin prick testing (SPT) and serum-specific IgE measurement, molecular and component-resolved diagnostic techniques are increasingly enabling more precise characterization of allergen sensitization patterns and supporting the development of precision medicine strategies (2).

Current management of HDM allergy includes allergen avoidance, pharmacotherapy with antihistamines and corticosteroids, and allergen immunotherapy (6). Although these approaches can effectively reduce symptoms, their long-term efficacy and disease-modifying potential remain variable. On the other hand, emerging biologic therapies targeting IgE and type-2 inflammatory pathways, including omalizumab and dupilumab, offer promising alternatives for patients with severe or treatment-refractory disease (7).

Beyond treatment, growing attention is being directed toward environmental interventions aimed at reducing indoor allergen exposure. Improvements in indoor air quality, humidity control, allergen-impermeable materials, and novel anti-mite technologies may complement medical management, although their effectiveness continues to be evaluated. Concurrently, advances in genomics, epigenetics, and microbiome research are revealing important interactions between genetic susceptibility and environmental exposures, opening new avenues for personalized prevention and therapeutic strategies (8).

In this context, I am pleased to present the Research Topic “Dust mite allergy: Immunological mechanisms, Environmental impact, Genetic susceptibility, Diagnosis, Therapeutic strategies & Preventive measures” which has been successfully published in “Frontiers in Allergy”. Working alongside Co-Editors Leticia Martín-Cruz and Pongsakorn Tantilipikorn, we assembled a Research Topic of articles addressing multiple dimensions of dust mite allergy. The Research Topic features two original research papers, one review article, and one brief research report, each contributing valuable insights into dust mite allergy.

Tamamoto-Mochizuki and Mishra in their brief research report investigated the role of neutrophils in a canine model of atopic dermatitis (AD) induced by HDM allergens. Dogs were exposed to HDM and skin biopsies were collected over a 96-hour period. Using histological and immunofluorescent analyses, it was found that neutrophils began infiltrating the skin between 24 and 96 h after allergen exposure with the highest increase in neutrophil numbers occurring at 48 h compared with baseline, suggesting that these cells may contribute to the early inflammatory phase and development of AD.

Expanding on these cellular pathways to explore specific therapeutic targets, the first original research in this topic by Chiang et al. examined the effect of the epidermal growth factor receptor (EGFR) inhibition in reducing inflammation caused by HDM allergen Der p II, a major trigger of allergic asthma. Human immune cells (peripheral blood mononuclear cells, THP-1 monocytes, THP-1-derived macrophages, and alveolar macrophages) were exposed to recombinant Der pII and then treated them with the EGFR inhibitors AZD-9291 and Tarceva (erlotinib). Der pII strongly increased the production of inflammatory mediators, including IL-6, IL-8, and TNF-α. Both EGFR inhibitors significantly reduced IL-6 and IL-8 secretion in immune cells. In THP-1-derived macrophages, AZD-9291 decreased IL-6 expression and reduced macrophage activation markers (CD14 and CD36). AZD-9291 also significantly lowered nitric oxide (NO) production in alveolar macrophages. Blocking EGFR with inhibitors such as AZD-9291 and Tarceva may help suppress HDM–induced allergic inflammation and could represent a potential therapeutic strategy for allergic asthma.

Liao et al. presented a prevalence study among 7,301 children with respiratory allergic diseases in Sichuan Province of China in the second original research of this topic. Sensitization was predominantly driven by HDM—especially *Dermatophagoides pteronyssinus* and *Dermatophagoides farinae*. Multiple-allergen sensitization was common and increased with age. Children with coexisting atopic diseases showed higher allergen sensitization rates, indicating a greater risk of progressive allergic disease. These findings highlight the importance of regional allergen patterns and support targeted allergen screening and personalized immunotherapy in pediatric patients.

Since allergen exposure patterns are highly dependent on geography, lastly, a narrative review by Tahir et al. illustrated the current understanding of HDM allergy in tropical regions, where warm and humid conditions allow mites to thrive year-round and contribute to high rates of allergic sensitization. The review focuses on the major mite species found in the tropics: *Dermatophagoides pteronyssinus, Dermatophagoides farinae* and *Blomia tropicalis.* The pattern of mite distribution and sensitization in tropical populations was highlighted. The paper compared diagnostic methods, including: SPT, Specific IgE testing and Component-Resolved Diagnosis (CRD), a molecular-based approach. The authors emphasized the need for region-specific research and diagnostic strategies because tropical regions have unique mite species and allergen cross-reactivity patterns. Future priorities include better molecular characterization of mite allergens and the development of standardized tropical mite extracts to improve diagnosis, immunotherapy, and public health interventions.

We would like to extend our sincere appreciation to all the contributors who participated in this Research Topic. It has been a rewarding experience to co-edit such a remarkable collection of scholarly works. Despite substantial progress, significant challenges remain. Existing therapies rarely provide sustained remission, allergen avoidance is difficult to achieve in real-world settings, and patient responses vary considerably owing to immunologic and genetic heterogeneity. Future research should therefore focus on highly sensitive molecular diagnostics, biomarker-guided precision medicine, novel immunomodulatory and biologic therapies, and innovative environmental control technologies. Such advances may ultimately transform the prevention and management of HDM-associated allergic diseases and improve long-term patient outcomes.
